# Psychological health and religious coping of Ghanaian women with infertility

**DOI:** 10.1186/s13030-017-0105-9

**Published:** 2017-07-12

**Authors:** Mabel Oti-Boadi, Kwaku Oppong Asante

**Affiliations:** 1 Faculty of Computing and Information Systems, Ghana Technology University College, Tesano, Accra, Ghana; 2 Department of Psychology, University of Ghana, P. O. Box LG 84, Legon, Accra, Ghana

**Keywords:** Women, Infertility, Psychological distress, Religious coping, Quantitative

## Abstract

**Background:**

Infertility has been shown to have considerable psychological effects on the well-being of couples, especially women. Religion has been found as a resource used by infertile women to cope with their distress. Little research has examined the influence of religious coping on psychological distress among infertile women in Ghana. This study examines the relationship between positive and negative religious coping and psychological health for women with infertility problems in Ghana.

**Methods:**

One hundred and fifty married women who were receiving assisted reproduction care in two specialized clinics were recruited for this study. Participants were administered with the Brief Symptom Inventory and Brief Religious Coping Scale to assess psychological health associated with infertility and religious coping respectively. A hierarchical regression was performed to examine the relative contribution of the domains of psychological health (i.e. somatization, anxiety and depression) in predicting negative religious coping and positive religious.

**Results:**

The results showed that negative religious coping was significant and positively correlated with somatization, depression and anxiety. Furthermore, a positive relationship also existed between positive religious coping and somatization and anxiety but not depression. After controlling for age and duration of infertility, somatization and anxiety predicted positive religious coping whilst all the domains of psychological health (somatization, anxiety and depression) precited negative religious coping.

**Conclusions:**

This study expanded on the existing literature by examining positive and negative religious coping with psychological distress associated with infertility for women. These results underscore the need for health professionals providing therapies for women with infertility to acknowledge and consider their religious beliefs as this influences their mental health.

## Background

Infertility is perceived as a major health related problem that affects people across all cultures and societies. Globally, it is estimated that about 10% of couples of reproductive age are unable to get pregnant or carry a pregnancy to term [1]. The incidence of infertility varies around the world and studies have shown that majority of those who suffer live in the developing countries in Sub-Saharan Africa where about 5 to 30% couples are infertile [1]. Despite Africa’s high population growth rate, prevalence of infertility in Africa is high and this raises a reason for concern as it comes across as a major reproductive health problem [2]. These statistics highlights the need for research in this unexplored area, as the experience and coping with infertility, has been shown to have considerable psychological effects on the well-being of couples, especially women [3, 4]. The psychological impact of infertility (e.g. depression and anxiety) has not received much attention particularly within the Ghanaian context.

In Ghana, infertility is fast becoming pervasive among the women population as an estimated 15% of women of childbearing age in Ghana experience infertility problems [5]. According to the Ghana Demographic and Health Survey [6], fertility rate among Ghanaian women has dropped from 6.4 children per woman in 1998 to 4.0 in 2008, making the trend the lowest in Sub-Saharan Africa. Infertility is generally defined as the failure to conceive after a year of regular sexual intercourse without contraception [7] and this is perceived as often caused by biological and other related factors in most western cultures [8]. However, in developing and underdeveloped countries, as the case may be in Ghana, infertility may be linked to supernatural causes, punishment from the gods for sins of the past, evil spirits, witchcraft and God’s retribution [9, 10].

In many cultures around the world motherhood is an important goal for most women [11, 12], and in the Ghanaian society, a high premium is put on childbearing among marriage couples, and the need for children is expressed in African maxims as ‘there is no wealth where there are no children’ [9]. Though many women around the world are currently choosing not to have children [13], voluntary childlessness is not common in Africa, as women who fail to bear children suffer humiliation and sometimes ridiculed and abused [9, 14, 15]. Research indicates that the experiences of infertile women prevents them from sharing with others what they go through [5, 16] and this further translates into high levels of stress and depression [4, 17–21].

Demographic variables such duration of infertility and age have been found to be associated with anxiety and depression among women with infertility problems. Duration of infertility has been found to increase stress among infertile women [3, 18]. Similarly, as age of infertile women increases, they tend to exhibit a higher susceptibility to depression [22–24].

Several empirical studies have revealed that women with infertility issues use religion to cope with their situation [2, 3, 5, 25], and that the use of religious coping strategies have been significantly linked with less infertility distress and fewer depressive symptoms [2, 25]. However, among other women, religion may increase their distress as they may use negative methods of religious coping such as being angry at God for their predicament [26, 27]. This suggests a relationship between religiosity and the psychological well-being of women with infertility problems [25, 26]. Research has shown that among women with infertility issues who are also religious, the experience of infertility goes beyond the medical experience since they take it as a spiritual crisis and a threat to the very core of their beliefs about self, life, and ultimate truths [27]. Religion plays an integral role in the life of Africans and Ghanaians especially when dealing with stressful situations [9]. Thus, exploring the use of religious coping with the inability to have children would contribute to the sparse research in Ghana and also contribute to academic literature. Religious coping refers to efforts made by individuals to deal with life stressors in ways that are associated with the divine or are imbued with divine-like qualities [28]. Religious coping strategies can be adaptive or maladaptive, hence, Pargament [28] categorized religious coping into two: positive and negative religious coping strategies. Positive religious coping generally rest on a secure relationship with whatever the individual may hold sacred whilst and negative religious coping are those that are reflective of tension, conflict, and struggle with the sacred [28].

Research conducted in Ghana, shows that infertile women experience severe psychological distress including depression, anxiety and stress [5, 14, 19, 20]. However, there are sparse literatures on the relationship between psychological distress and religious coping, taking into consideration that religion plays an integral part of life of Ghanaians in general. The study was therefore conducted to examine the relationship between the psychological distress and the use of religious coping among a cross-section of Ghanaian women undergoing fertility treatment in Ghana. We hypothesized that women facing fertility problems would experience higher levels of psychological distress. There would also be a positive significant relationship between duration of infertility, age and psychological distress (anxiety, depression and somatization). There would be a negative relationship between positive religious coping and psychological distress on one hand, and a positive relationship between negative religious among women with fertility problems on the other hand. The findings of the study would contribute to existing knowledge in the research area of religious coping and infertility. Additionally, the findings from this study would help in the development of appropriate mental health and community interventions programmes for women experiencing psychological distress as a result of their infertility. Depression as used in this study refers to low moods that interfere with behaviour and general activity, and somatization refers to physical symptoms that are caused by psychological or emotional factors such as anxiety and depression.

## Methods

### Sample and procedure

A cross-sectional survey was conducted in 2013, involving 150 women with fertility problems who were purposively sampled among other patients attending two specialized assisted reproduction clinics in Accra, Ghana. Participants were included in the study if they fulfilled the WHO definition of infertility which states that the inability of a sexual active non-contraceptive using woman to have a live birth after 12 or more months of regular sexual intercourse without a male factor. Thus, women with both primary infertility and secondary infertility problems were included in the study. Primary infertility as used in this study refers to the inability of a sexually active, non–lactating and non-contraceptive using woman to have a live birth after 12 or more months of regular sexual intercourse, and secondary infertility refers to women who are having the difficulty to have a live birth or hold pregnancy for full term, even though they had previous had a live birth. Women who had male factor infertility were excluded.

After approval was given by the hospital administration, the study was introduced to the participants. Those who agreed to participate in the study gave a written informed consent. Each questionnaire packet included a letter from the researchers explaining the purpose and procedures for the study. The cover page of the questionnaire briefly explained the aims of the study and provided instructions to the respondents on how to fill it up. It also provided information about the researchers. It also mentioned that anonymity and confidentiality would be maintained, and that participation in the study was voluntary. Participants were clearly informed that they could stop participating in the study at any time without any consequences to them. It also specified that data collected from the study would be used only for research purposes. The data collection period lasted for 10 weeks. Ethical Clearance for the study was given by the Ghana Technology University College and the Administrators of the two fertility clinics.

### Measures

Data was collected by means of a self-administered structured questionnaire which includes questions relating to sample demographics as well as questions on religious coping and psychological distress.

#### Demographic items

A socio-demographic questionnaire was designed by the authors to elicit information on socio-demographic variables including participant’s age, level of education, employment status and duration of infertility.

#### The Brief Symptom Inventory (BSI 53)

The BSI is a 53-item inventory [29] designed to assess psychological systems of psychiatric, medical, and normal individuals, was used to measure psychological health. Responses are provided using a 5-point Likert scale that ranges from 0 = not at all to 4 = extremely true. The inventory assesses nine categories of psychological symptoms (somatization, obsession-compulsion, interpersonal sensitivity, depression, anxiety, hostility, phobic anxiety, paranoid ideation and psychoticism). For the purpose of this study only 3, subscales (somatization, depression and anxiety) were used. Each subscale of the BSI was calculated by adding up the responses on the items under each subscale and then dividing the total by the number of items. Validity and reliability of the instrument have been found to be adequate and reported internal consistency estimates ranging from 0.71 to 0.98 across the nine dimensions [29]. The Cronbach alpha reliability coefficient in this study is 0.98.

#### Brief RCOPE

The Brief Religious Coping Scale (Brief RCOPE), a self-report instrument was used to measure patterns of positive and negative religious coping, using seven items each [30]. Positive religious coping strategies include spiritual connection, seeking spiritual support, religious forgiveness, collaborative religious coping, benevolent religious appraisal, religious purification, and religious focus. Negative religious coping strategies are spiritual discontent (two items), punishing God reappraisal (two items), interpersonal religious discontent, demonic reappraisal, and reappraisal of God’s power. The individual items on positive and negative coping were combined to yield one single summary score of positive and negative coping, respectively. The Brief RCOPE has shown good psychometric properties in a variety of different samples [31, 32]. The present study found values of 0.73 and 0.71 for the positive and negative religious coping scale, respectively.

### Statistical analysis

Data analyses were conducted using Statistics Package for Social Sciences (SPSS) version 21.0. Descriptive statistics were used to examine the demographic characteristics of the sample. Pearson correlation coefficients matrix was computed to examine the associations among the variables, especially the subscales of Brief RCOPE, somatization, anxiety and depression. A hierarchical regression was performed to examine the relative contribution of the domains of psychological health (i.e. somatization, anxiety and depression) in predicting negative religious coping and positive religious. Following the entry of demographic variables (age and duration of infertility), the next block of variables (somatization, anxiety and depression) was entered into the model. This procedure was followed to ensure that those variables that were strongly associated with negative and positive religious coping would account for unique variance beyond that accounted for by the demographic variables. Information is provided for the standardized beta weight (β), the *R* ^2^ and the ∆R^2^ accounted for in the model. All statistical tests were performed using two-tailed, and a *p* < 0.05 was considered statistically significant.

## Results

### Sample characteristics

The participants included a total of 150 women undergoing fertility treatment in Accra, Ghana (Table 1). This represents a response rate of 85% (only 150 out of 176 agreed to take part in the study). Their ages ranged from 27 to 44 years (*M* = 34.3; *SD* = 5.67) and approximately (67%) were in their 30s. Over half (53.3%) have had tertiary education and a large majority of the women, 86.7% were employed. The duration of infertility was found to 3–15 (*M* = 5.8; *SD* = 3.4) years.

**Table 1:** Demographic Characteristics of the study population (*n* = 150)

Characteristics		N (%) or Mean (SD)
Mean age in years		34.3 (5.67
Ages (n, %)		
	< 30	35 (23.3)
	30 –35	52 (34.7)
	36 – 39	13 (8.7)
	40 – 44	50 (33.3)
Mean of infertility in years		5.8 (3.4)
Duration of Infertility		
	1–5	91 (60.7)
	6 – 10	18 (12.0)
	11– 15	41 (27.3)
Education		
	Junior Secondary	20 (13.3)
	Senior Secondary	48 (32.0)
	Tertiary	82 (54.7)
Employment Status		
	Employed	130 (86.7)
	Unemployed	20 (13.3)

### Relationship among variables in the study

The results presented in Table 2 shows the relationship between age, duration of infertility, and psychological health. The findings age was positively associated with duration of infertility (*r* = 0.784, *p* < 0.001), somatization (*r* = 0.687, *p* < 0.001), depression (*r* = 0.241, *p* < 0.01) and anxiety (*r* = 0.313, *p* < 0.001). This suggests that infertility among women increases with age. The results also imply that psychological distress increases as women with infertility grow older.

**Table 2:** Relationship between age, duration of infertility, religious coping and psychological distress

Variables	1	2	3	4	5	6
1. Age	1	--------	--------	--------	--------	--------
2. Duration of infertility	.784***	1	--------	--------	--------	--------
3. PRCOPE	.258***	.168*	1	--------	--------	--------
4. NRCOPE	.342*	.220***	.234**	1	--------	--------
5. Somatization	.687***	.416***	.381***	.501***	1	--------
6. Depression	.241**	.222**	.116	.481**	.832***	1
7. Anxiety	.313***	.067	.244**	.380***	.862***	.969***

*PRCOPE* Positive religious coping, *NRCOPE* Negative religious coping

*p < .05; **p < .01; ***p < .001

The result as presented in Table 2 also shows that positive religious coping was significantly related to somatization (*r* = 0.381, *p* < 0.001), and anxiety (*r* = 0.244, *p* < 0.01). This implies that women who use positive religious coping strategies are more likely to have high level of anxiety and somatization symptoms. Findings also revealed that negative religious coping was significant and positively correlated with age (*r* = 0.342, *p* < 0.05), duration of infertility (*r* = 0.220, *p* < 0.001), somatization (*r* = 0.501, *p* < 0.001), depression (*r* = 0.481, *p* < 0.001) and anxiety (*r* = 0.380, *p* < 0.001). This result suggests that women with infertility problems who uses negative religious coping are more likely to have elevated levels of somatization, depression, and anxiety.

### Predictors of positive and negative religious coping

Results of the hierarchical regression analyses are summarized in Table 3. When positive religious coping was considered as an outcome variable, only age (*β* = 0.37, *p* < 0.05) was found to be a significant predictor in model 1 and accounted for 9.1% of the variance in positive religious coping; *F*(2, 117) = 5.83, *p* < 0.01. In model 2, in which the domains of psychological health (i.e. somatization, anxiety and depression) was examined, statistically significant predictors for somatization (*β* = 0.58, *p* < 0.05) and anxiety (*β* = 0.61, *p* < 0.05) were found: The regression model accounted for 14.5% of the variance in positive religious coping ∆*R* ^2^ = 0.145, *F*(2117) = 4.8, *p* < 0.001.

**Table 3:** Summary of hierarchical egression models results

	PRCOPE		NRCOPE	
	Total *R* ^*2*^ = 0.091		Total R^2^ = 0.075	
	β	*∆R* ^*2*^	β	∆R^2^
Step 1		0.145		0.218
Age	0.37*		0.11	
Duration of infertility	0.09		0.28*	
Step 2				
Age	0.48*		−0.14	
Duration of infertility	0.11		0.33*	
Somatization	0.58*		0.82**	
Anxiety	0.61*		0.61**	
Depression	-----		0.69**	

*PRCOPE* Positive religious coping, *NRCOPE* Negative religious coping

*p < .05; **p < .01

When negative religious coping was considered as an outcome variable, only duration of infertility (*β* = 0.28, *p* < 0.05) was found to be a significant predictor in model 1 and accounted for 7.5% of the variance in negative religious coping; *F*(2, 117) = 4.8, *p* < 0.05. In model 2, in which the domains of psychological health was examined, statistically significant predictors for all the domains were found: somatization (*β* = 0.82, *p* < 0.01), anxiety (*β* = 0.61, *p* < 0.01) and depression (*β* = 0.69, *p* < 0.05). The regression model accounted for 21.8% of the variance in negative religious coping ∆*R* ^2^ = 0.218, *F*(2117) = 6.2, *p* < 0.001.

## Discussion

The purpose of this study was to examine the relationship between religious coping strategies and psychological distress associated with infertility among women in Ghana. Few studies have investigated the relationship among these variables within the Ghanaian context. The results showed that negative religious coping was significant and positively correlated somatization, depression and anxiety. Furthermore, a positive relationship also existed between positive religious coping and somatization and anxiety but not depression. Our findings also revealed that psychological distress (somatization, depression and anxiety) increase with age of women and duration of infertility.

The Brief RCOPE scale [30] in this study had acceptability reliability coefficient which is similar to studies conducted by Gardner et al. [31] with university students in New Zealand and Pearce, Singer and Prigerson [32] with caregivers of cancer patients in the United States. Despite its reliability, responses to individual items on both negative and positive coping dimensions showed significant floor and ceiling effects. Negative skew and ceiling effects on the positive religious coping scale have also been reported in other studies using the Brief RCOPE [32]. For example, Khan and Watson [33] found that the average score of positive religious coping responses was 3.01 (SD = 0.59), out of a highest possible score of 4.00, a value similar to the average score in this study. Overall, these results suggest that women with infertility problems uses high levels of positive religious coping strategies and low levels of negative religious coping.

This study found that Ghanaian women with fertility problems experience high levels of psychological distress. This observation is consistent with a myriad of previous findings which have consistently found that women with fertility problems experience higher levels of psychological distress [4, 15–18]. A previous study in Ghana showed that women with infertility problem do not share their problems with others as a result of stigma [5]. Their inability to open up to others could be a contributing factor to the elevated levels of psychological distress in this study. The study also revealed a positive significant relationship between duration of infertility, age and psychological distress (anxiety, depression and somatization). Higher levels of psychological distress were thus associated with aging, suggesting that older women were more likely to experience higher levels of psychological distress than younger women. This means that as a women grows old she is more likely to experience distress. This finding is consistent with studies conducted elsewhere [22, 23]. A plausible explanation for this observation is that many women perceive their biological clocks to be ticking as they wait to have children and may likely experience distress as they do not know when they will have a child.

Duration of infertility was also positively associated with higher levels of depression, somatization and anxiety among women with infertility problems in the current study. This finding is consistent with previous studies [3, 18, 34, 35]. On the contrary, other studies [22, 35] have found that the longer the period of infertility, the lower the levels of anxiety and depression experienced by infertile women. It could be explained that as women with infertility problems think of how long it is taking them to bring forth their own children, they experience elevated level of psychological distress, and especially in an African context where having your own children is seen as a validation of marriage [9]. Further, during the early stages of being diagnosed with infertility, the hopefulness of the woman for a successful outcome of medical intervention is higher, but as the intervention progresses without a success combined with the stress of moving from one hospital to the other, they may become psychologically stressed up with fading hopes of conception [19].

Unexpectedly, the results indicated that positive religious coping was predicted by both somatization and anxiety. This finding is inconsistent with existing general consensus in literature where positive religious coping is often associated with a reduction in depressive symptoms in infertile women [2, 3, 5, 25]. Plausible explanations for such discrepancies could be attributed to the measures used in the study and cultural differences. Probably, some items found in the religious coping measure are not culturally sensitive to Ghanaian women. Further, women with infertility may also experience high levels of depression and anxiety either because their prayers are not being answered by God or whether going for fertility treatment will be endorsed by their religion. Religious women could also experience heightened distress from their religious communities which emphasizes on childbearing as the ultimate outcome of marriage and this could lead to the assertion that religious beliefs may increase the psychological distress of infertile women [30]. It is also like that these participants may have used high levels of positive religious coping as and when their stress levels increased. Thus, in their attempt to respond to these levels of psychology distress, they may engage in greater religious coping.

The findings of the study also showed that negative religious coping was associated with increasing levels of anxiety, depression and somatization. This outcome was not surprising, as negative religious coping has been associated with poor mental health such as depression, anxiety, PTSD symptoms, pain and negative affect [30]. In Ghana and in most part of Africa, religion plays a very important role in the interpretation and experience of problems, and a woman’s inability to give birth within the first year of marriage may likely affect her religious beliefs. It is likely that participants might have questioned the power of God or may have perceived their situation as punishment from God. Given the socially undesirable nature of negative religious coping in the context infertility among women, it is especially noteworthy that the use of negative religious coping was related to poor psychological health.

### Limitations and future directions

This study was beset with some limitations. First, the small sample size was collected using purposive sampling of clinical based participants, an indication that the results are not generalizable to all women with infertility problems in Ghana. Future studies investigating psychological health among women should include a large sample size and infertile women in the general population as responses from those in specialized clinics may be prone to sampling bias. Second, the cross-sectional design used rules out the possibility to determine any cause-and-effect relationship between religious coping and psychological distress. Third, the measures used have not been used in Ghana. However, they were chosen based on evidence of reliability and validity in infertility research whenever possible. Future research will need to revisit the wording of the Brief RCOPE items to avoid ceiling and floor effects. Fourth, the survey was based on self-report, which were likely to be biased. Knowing their medical history would have helped to explain their psychological health problems. Finally, future research should use a qualitative approach to explore how women with fertility problems to ensure greater insight into this phenomenon and to understand better why infertile women are more likely to employ religious coping strategies. It may also be interested to explore whether religiosity of infertile women increases their psychological distress.

## Conclusion

The present study explored the relationships between psychological distress and positive and negative religious coping among women with infertility problems in Ghana. The results showed that psychological distress (somatization, depression and anxiety) in women with infertility increases with aging and duration of infertility. The results also showed that negative religious coping was significant and positively correlated somatization, depression and anxiety. Furthermore, a positive relationship also existed between positive religious coping and somatization and anxiety but not depression. This is the first empirical study to examine women with infertility using both positive and negative religious coping strategies to deal with the psychological challenges associated with infertility. Religiosity appears to play a significant role in the lives of Ghanaian infertile women. These results call attention to the need for more empirical research on the psychological distress and religious coping link. Health professionals providing therapies for women with infertility should acknowledge and consider their religious beliefs as this influences their mental health.
